# No effect of a commercial carbohydrate‐menthol drink on thermal perceptual measures or 15‐min time trial performance compared to commercial carbohydrate drink in hot humid conditions

**DOI:** 10.1002/ejsc.12037

**Published:** 2024-02-26

**Authors:** Patrick W. Bray, Sam D. Blacker, Andrew T. West, Tessa R. Flood

**Affiliations:** ^1^ Institute of Sport Nursing and Allied Health University of Chichester Chichester UK; ^2^ Department of Sport and Exercise Science Institute of Sport Manchester Metropolitan University Manchester UK

**Keywords:** environmental physiology, nutrition, performance, physiology

## Abstract

This study assessed the effect of a commercial carbohydrate menthol drink on cycling time trial (TT) performance in hot and humid conditions compared with a carbohydrate only drink. Ten participants (5 women; V̇O_2max_: 52.3 ± 8.6 mL kg^−1^ min^−1^, Peak Power Output: 286 ± 56 W) completed a 40‐min cycling preload (50% V̇O_2max_) followed by a 15‐min self‐paced TT in hot (∼35°C) and humid (∼54%) conditions on two occasions (double blind, crossover design). Every 10‐min, 85 mL of carbohydrate (CHO; SIS GO Energy, 60 g h^−1^) or carbohydrate and menthol (CHO + MEN; SIS Turbo+ 60 g h^−1^, 0.01% menthol) was swilled (∼10‐s) and ingested. Rectal temperature (*T*_rec_) and heart rate (HR) were recorded throughout. Thermal sensation (TS), thermal comfort (TC) and rating of perceived exertion (RPE) were recorded every 5‐min. Taste and aftertaste were rated from very pleasant (+5) to very unpleasant (−5). TT performance (total work; kJ) was similar between CHO (153 kJ [95% CI: 129–177 kJ]) and CHO + MEN (151 kJ [128–178 kJ]). During preload exercise, *T*_rec_ increased by ∼0.9°C and was similar at the end of the TT (∼38.20°C). Mean preload HR was ∼140 b min^−1^ in each condition and reached ∼177 b min^−1^ at the end of the TT. TC was rated as ‘*much too warm*’ and TS rated as ‘*very hot*’ in both conditions. Both conditions were ‘*extremely hard*’ (end point RPE ∼19). All participants preferred the taste and aftertaste of the CHO drink. The commercial carbohydrate menthol drink offered no additional ergogenic benefit compared to a carbohydrate only drink during cycling exercise performed in hot and humid conditions.

## Highlights

- The addition of menthol to a carbohydrate drink had no effect on 15‐min time trial performance.
- The addition of menthol to a carbohydrate drink had no effect on thermal perceptual measures or physiological measures.
- Taste and aftertaste perceptions were favourable towards the carbohydrate drink compared to the carbohydrate‐menthol drink.

## 1 INTRODUCTION

Menthol (L‐menthol) has been shown to improve thermal comfort (TC) and sensation during endurance performance in the heat, leading to performance improvements when swilled and/or consumed orally (Jeffries & Waldron, 2019). Menthol elicits a cooling sensation by stimulating the transient receptor potential melastatin 8 (TRPM8), which are found in various locations around the body (Steinritz et al., 2018). Research has targeted TRPM8 in the oral cavity (mouth rinse/drink) or the skin (topical application) (Gillis et al., 2010; Jeffries & Waldron, 2019; Schlader et al., 2011). However, it is postulated that oral application could induce a faster and greater response to menthol due to the reduced thickness of the stratum corneum in the oral cavity, and thus potentially promote greater activation of oral TRPM8 receptors (Crosby et al., 2022). The TRPM8 receptors evoke a signal that travels to the hypothalamus and somatosensory cortex (Andersen et al., 2014) via the trigeminal nerve (Best et al., 2021). This elicits a cool sensation, reducing perceived thermal sensation (TS) and discomfort, which alter perceived effort of exercise to improve performance (Flouris & Schlader, 2015; Jeffries & Waldron, 2019; Schlader et al., 2011).

This notion is supported by a meta‐analysis, which highlighted the link between athletes feeling cooler in hot conditions and beneficial performance changes (e.g. increase in exercise duration and/or intensity) (Jeffries & Waldron, 2019). Fluids with a 0.01% menthol concentration have been found to reduce TS, increasing an athletes exercise capacity and thus improving performance when swilled orally in hot and humid conditions (Flood et al., 2017; Gavel et al., 2021; Jeffries et al., 2018; Mündel & Jones, 2010; Stevens et al., 2017). Gavel et al. (2021) highlighted an increase of 6% in mean power output and a 2.3% improvement in 30 km time trial (TT) cycling performance following oral menthol rinsing. Additionally, de Camargo et al. (2022) reported a 2.3% reduction in 10 km running time following menthol rinsing compared with no intervention. The reductions in TS have been suggested to pose a risk to athletes, as a reduced sensation of one's thermal state could lead to athletes developing exertional heat illness (Stevens & Best, 2017); however, an expert consensus statement determined menthol was a safe supplement when administered correctly that could be used in elite competition (Barwood et al., 2020). Furthermore, Best (2022) recently determined liquid doses between 0.1 and 0.5 g L^−1^ have been safely utilised. Drinking was selected over just rinsing the fluid to attenuate dehydration in the heat as well as aim to stimulate receptors found in the upper oral cavity. Also, supplement selection was guided by availability on the market at the time of data collection.

During endurance events in hot conditions, it is unlikely that menthol would be used in isolation, with athletes likely to also be consuming other sports foods and supplements simultaneously. It is well documented that carbohydrate consumption increases carbohydrate oxidation during exercise (Burke, 2001) and improves exercise performance (Stellingwerff & Cox, 2014). Recommendations of consuming 60–90 g h^−1^ carbohydrate during endurance exercise are widely reported for maintaining optimal endurance exercise capacity (Burke, 2021; Jeukendrup, 2011). Therefore, in applied settings, it is highly likely that menthol supplements would be used during prolonged exercise (≥1‐h) alongside ingestion of carbohydrate. Additionally, co‐ingestion of menthol with carbohydrate also provides a more practically convenient solution for athletes to consume the required carbohydrates alongside the menthol supplement.

An added intricacy of consuming menthol and carbohydrate concurrently is that rinsing the mouth with carbohydrate alone has been shown to improve exercise performance (Chambers et al., 2009) with effects dependent on rinse timing, duration and concentration (Best et al., 2021). It is postulated that the physiological and psychological responses associated with carbohydrate mouth rinsing stem from oral receptor activation of the central nervous system (Best et al., 2021; Rollo & Williams, 2011). Studies investigating carbohydrate rinsing have shown activation of type 1, member 2 and 3 receptors (T1R2, T1R3) in the oral cavity that stimulate reward areas in the brain via dopamine paths (Chambers et al., 2009; Lee & Owyang, 2017), leading to a potential improvement in exercise intensity via improvement in motivation. Therefore, the effectiveness of carbohydrate rinsing is influenced by multiple factors (e.g. nutritional state, carbohydrate concentration and rinse duration) (Best et al., 2021). There are different outcomes regarding carbohydrate rinse effectiveness with numerous studies showing improvements in performance in warm conditions (Best et al., 2021; Lane et al., 2013; Sinclair et al., 2014). However, contradictory findings have also been observed. One paper found no improvement with carbohydrate rinsing in the heat, which was attributed to greater physiological stress alongside increased subjective measures from elevated thermal strain (Cramer et al., 2015). Additionally, Kamaruddin et al. (2023) found carbohydrate rinsing to be no more effective than placebo rinsing on endurance running in warm and humid conditions.

To date, relatively few studies have combined menthol and carbohydrate and assessed the effect on performance. Notably of these, one paper assessed isolated swilling in the heat and highlighted greater sensations in oral cooling following menthol swilling, alongside improved TT performance with carbohydrate swilling (Best et al., 2021). A second study assessed a commercially available carbohydrate menthol drink in a thermoneutral environment but found no improvement in exercise capacity (Podlogar et al., 2021). Therefore, the aim of the current study is to compare two commercially available drinks; a carbohydrate only (CHO) and a carbohydrate and menthol (CHO + MEN) drink, on physiological and perceptual measures to a 40‐min sub‐maximal pre‐load and 15‐min TT performance in hot and humid conditions. The present study will progress this field by building on previously published work for further understanding of carbohydrate and menthol co‐consumption during exercise. As such, this study hypothesised that work done TT performance would improve when supplementing with CHO + MEN drink compared with the CHO drink.

## 2 METHOD

### 2.1 Participants

Ten (*n* = 5 women) moderately trained participants (Tier 1/2 [McKay et al., 2022] mean ± standard deviation; age: 23 ± 5 years, stature: 176.0 ± 7.7 cm, body mass: 74.5 ± 11.3 kg, body fat: 17.9 ± 6.2%, maximum aerobic capacity [V̇O_2max_]: 52.3 ± 8.6 mL kg^−1^ min^−1^ and peak power: 286 ± 57 W) volunteered. Participants provided written informed consent and completed a health history questionnaire before starting the study. Participants were excluded if they had (1) travelled to a hot country in the last 3 months, (2) had participated in heat acclimation and (3) took any supplements known to impact thermoregulation or substrate utilisation, that is, polyphenols. One woman was using a monophasic combined oral contraceptive pill (Lucette) and was tested in the pill‐phase. Four women were eumenorrheic, and both testing sessions occurred in the same phase of the menstrual cycle (follicular [before ovulation]; *n* = 3, Luteal [after ovulation]; *n* = 1). The study received ethical approval from the University Research Ethics Committee (#2122–1800012).

### 2.2 Study design

A randomised double‐blind crossover design was adopted whereby participants completed two experimental trials (60‐min) in hot and humid conditions (35.0°C, 54% RH) consuming either a carbohydrate (CHO) (SIS GO Energy, Lemon flavour) or carbohydrate‐menthol (CHO + MEN) (SIS Turbo+, Citrus flavour) drink. Drinks were provided in identical closed top bottles to conceal smell and colour with the bottles placed inside the environmental chamber 20 min prior to the start of testing. All drinks were made up by a staff member who remained external to the project (full drink delivery protocol described below in experimental protocol below). Participants were blinded to the true aim of the study, and instead were told we were assessing favours in carbohydrate drinks. No mention of menthol was made to participants to minimise bias in flavour. Drinks were made up by a member of staff who remained external to the research project. Drinks were matched to ensure participants received the 60 g h^−1^ carbohydrate recommendation for endurance exercise under both experimental conditions (Vitale & Getzin, 2019).

### 2.3 Preliminary visit

Participants stature (Holtain Counter, Holtain Ltd.), body mass (Seca model 873; Seca Ltd.) and body fat (Tanita BC–418MA, Tanita EU) were measured. First, a sub‐maximal assessment was completed to estimate a work rate equating to 50% V̇O_2max_ for each participant. Participants completed four submaximal 5‐min stages, beginning at 60 W and increasing by 20 W each stage and was completed on an electromagnetically braked ergometer (Lode Excalibur, Excalibur Sport, Lode). Expired gas samples were collected in the last 90 s of each 5‐min stage using the Douglas Bag technique. These were analysed using a Servomex gas analyser (Servomex 5200; Servomex) calibrated prior to each trial with known gas concentrations. Volume was determined via a Harvard dry gas metre (Harvard Apparatus) alongside temperature (NS 920; Hanna Instruments). Haldane transformations corrected for F_i_O_2_ and F_i_CO_2_ were used to calculate V̇O_2_ and V̇CO_2_. In addition, heart rate (HR) (Polar RS800; Polar Electro UK Ltd.) and rating of perceived exertion (RPE) (Borg, 1982) were measured at the completion of each stage completion. Following a 15‐min rest, participants completed a step based maximal oxygen uptake (V̇O_2max_) test, which began at 120 W and increased by 20 W per minute until exhaustion. Expired gas samples (∼60‐s) were collected via Douglas bags once HR was above 150 b min^−1^, and V̇O_2max_ was determined as the highest value observed during the test, alongside RER >1.05 and HR within 10 b min^−1^ of age‐predicted maximum. Peak power (W) was recorded as the work rate of the final stage completed. Following the V̇O_2max_ assessment, linear regression (ordinary least squares) was used to describe the relationship between V̇O_2_ and power. Subsequently, the work rate (W) that would require 50% V̇O_2max_ was calculated. This then informed the derivation of each participants individualised linear factor for the work completed time‐trial by utilising Equation (1) (Jeukendrup et al., 1996).

Following 30‐min of rest, participants completed a 15‐min maximum work completed time‐trial to familiarise them to the experimental test protocol and drinking/swilling protocols (Flood et al., 2020).

Equation (1). Calculation of linear factor (Jeukendrup et al., 1996).

(1) $$\text{Linear}\;\text{factor}=50\%\;\text{power}÷{\text{cadence}}^{2}$$

### 2.4 Physical activity and dietary controls

Participants were asked to refrain from strenuous exercise, caffeine and alcohol 24‐h prior to any testing session. Participants arrived to the laboratory future and completed self‐reported food diaries 24‐h prior to experimental session 1 and were instructed to replicate this diet prior to session 2. Dietary analysis was conducted in Nutritics (Nutritics 2019; Research Edition v1.1), providing overall intake of energy (kcal) and carbohydrate, fat and protein (g).

### 2.5 Experimental protocol

**FIGURE 1:**
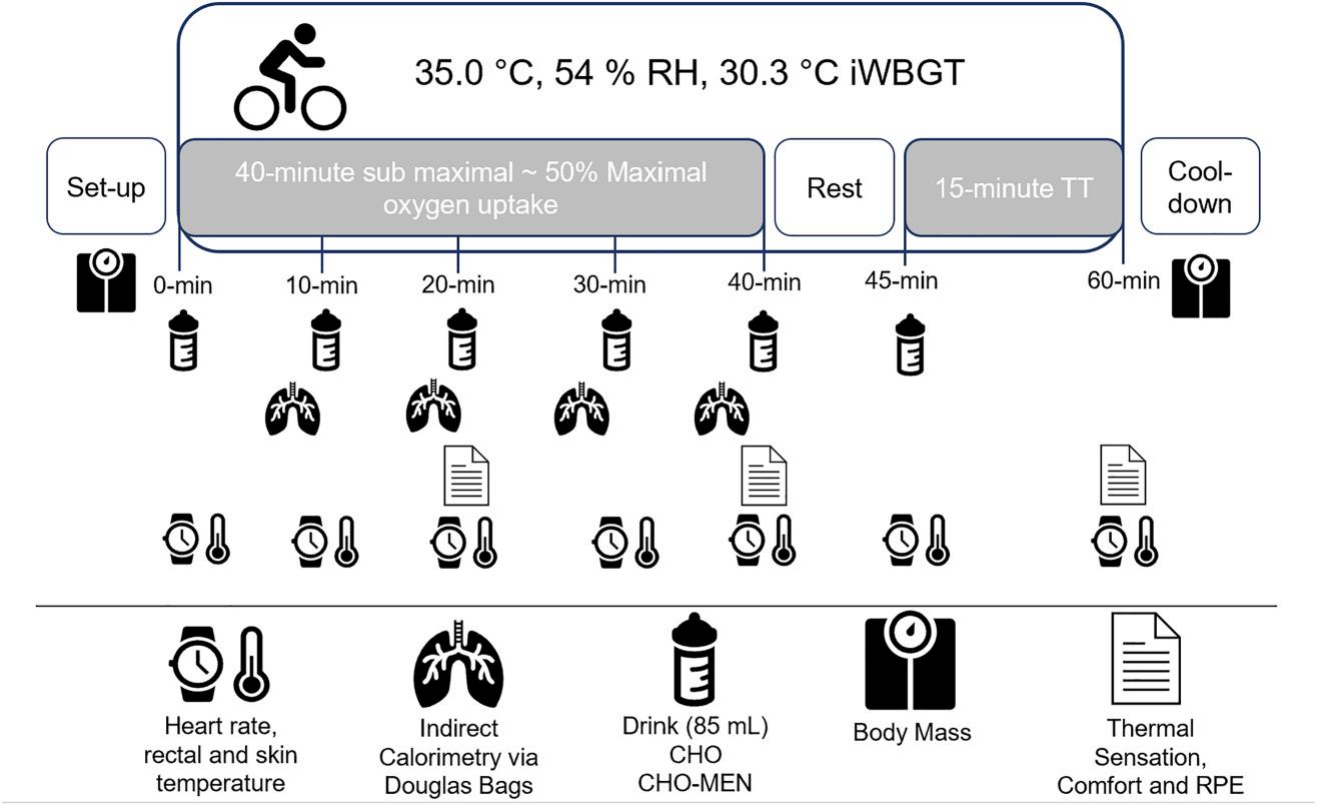
Experimental trial protocol schematic. Heart rate, rectal and skin temperatures were logged throughout the trial but presented as 0, 20, 40 and 60‐min.

Participants completed testing at the same time of day (±1‐h [either morning or afternoon]). Figure 1 shows the experimental protocol design. All experimental sessions occurred in an environmental chamber (TISS Model 201003‐1; TISS Services UK) with environmental conditions recorded using a wet bulb globe temperature metre (Kestrel 5400; Kestrel Meters). Euhydration was determined using urine osmolality <700 mOsm kg (Sawka et al., 2007) (Osmocheck PAL‐OSMO, Vitech Scientific), and clothed body mass was recorded. All testing was completed with participants clothed in shorts and a sports shirt (female participants wore a sports bra in addition to the sports shirt).

Participants self‐inserted a rectal thermistor (Edale Instruments) 10 cm past the anal sphincter for measurement of deep body temperature. Skin temperature thermistors (ELEUS‐T‐NL‐0303; Eltek Ltd.) were attached at 4 sites (neck, shin, scapula and hand) and were used to calculate mean skin temperature using the International Standards Organisation (ISO, 2004) weighted equation (mean skin temperature = [neck × 0.28], + [scapular × 0.28] + [hand × 0.16] + [shin × 0.28]). Rectal and skin temperature were logged using GenII Eltek transmitters and Eltek Squirrel 1000 series (Eltek Ltd.) in 20‐s intervals. Mean body temperature was calculated using rectal and skin temperature (Lenhardt & Sessler, 2006). HR was recorded continuously at 5‐s intervals via a short‐range telemetry (Polar RS800; Polar Electro UK Ltd.), and physiological strain index (PSI) was calculated using maximum HR from the V̇O_2max_ assessment and the rectal temperature upper limit of 40.0°C (Byrne & Lee, 2018).

Participants then entered the environmental chamber and completed 5‐min rest before completing 40‐min cycling at 50% V̇O_2max_ (103 ± 29 W). At the beginning and every 10‐min during the protocol, the participants consumed either CHO or CHO + MEN in 85 mL boluses. Each drink bolus was split into part A (25 mL, 10‐s mouth rinse and drink), followed by part B (60 mL drink bolus), which was consumed within 30‐s of consuming part A (total volume consumed 510 mL). Expired gas collections were completed every 10‐min using Douglas Bags. These were analysed after each session and corrected for FiO_2_ and FiCO_2_ (Betts & Thompson, 2012). Carbohydrate and fat utilisation were calculated using standardised equations (Péronnet & Massicotte, 1991). Perceptual measurements (RPE (Borg, 1982), TC (Bedford, 1936)) and TS (ASHRAE, 2004) were recorded every 5‐min.

Following completion of the 40‐min of exercise, participants rested on the bike for 5‐min. The ergometer was set to linear mode and the individual linear factor applied. The final bolus of drink was provided 60‐s prior to the TT start; participants then completed the 15‐min TT. Participants were blinded to work output and cadence and only given elapsed time at standardised intervals (5‐min, 10‐min, 12‐min, 13‐min, 14‐min and then at 15‐s intervals during the final minute). On completion of the TT, participants immediately vacated the chamber where excess sweat was removed and post exercise mass recorded.

Following completion of the last trial, a questionnaire was used to assess the performance of both drinks. Participants were asked to pick the preferred drink, and then taste, aftertaste, feeling hot/cold and overall experience were rated on a 100 mm visual scale from −5 to 5 (Podlogar et al., 2021).

All data were analysed using JASP (JASP [Version 0.16.0]). Physiological data were presented as mean (95% confidence interval). All 20‐s logged data (HR, rectal and skin temperature) were visually inspected before values were identified at 0, +20, +40 and +60‐min from the raw data. Analysis on perceptual data were conducted on ratings at selected time points of 0, +20, 40 and +60‐min.

All data were tested for normality before analysis. Equality of conditions data were analysed using paired *t*‐tests, and physiological and thermoregulatory data were analysed using a repeated measures ANOVA with fixed factors for condition (CHO, CHO + MEN) and time (0‐min, +20‐min, +40‐min and end‐TT). To provide the reader with an objective indication of the magnitude of the differences, effect sizes were calculated as Cohen's *d* for *t*‐tests or as partial eta squared ($\eta_{p}^{2}$) for RM‐ANOVA. For reference, values of 0.2, 0.5 and 0.8 correspond to small, medium and large effect sizes for *d,* respectively, and values of 0.01, 0.09 and 0.25 are considered to be small, medium and large effect sizes for $\eta_{p}^{2}$, respectively (Cohen, 1988). Perceptual data were presented as median (range) and were analysed using non‐parametric Friedman tests, and taste perceptions were analysed using a Wilcoxon signed rank test.

## 3 RESULTS

### 3.1 Equality of conditions

Supplementary data were provided for the equality of conditions data. There were no differences between the two trials for environmental temperature, humidity, hydration status and dietary intake. Dietary intake data was only analysed for 8 of the 10 participants due to errors in reporting.

### 3.2 Physiological and thermoregulatory responses

The key physiological and thermoregulatory results are shown in Table 1 for 20 min, 40 min (end of sub‐max cycling) and end of TT (60 min).

**TABLE 1:** Physiological and perceptual variables for the CHO and CHO + MEN conditions.

Variable	Condition	Baseline	20‐min	40‐min	End of TT	ANOVA (condition, interaction)
Rectal temperature (°C)	CHO	37.00 (36.67, 37.32)	37.47 (37.22, 37.72)	37.87 (37.59, 38.14)	38.27 (38.04, 38.49)	*F*_(1,9)_ = 0.072, *p* = 0.795, $\eta_{p}^{2}$ = 0.008
	CHO + MEN	37.00 (36.82, 37.17)	37.45 (37.24, 37.65)	37.85 (37.55, 38.14)	38.24 (37.99, 38.49)	*F*_(3,27)_ = 0.071, *p* = 0.975, $\eta_{p}^{2}$ = 0.008
Skin temperature (°C)	CHO	33.76 (33.07, 34.45)	35.22 (34.83, 35.62)	35.21 (34.76, 35.65)	35.28 (34.71, 35.84)	*F*_(1,9)_ = 2.504, *p* = 0.148, $\eta_{p}^{2}$ = 0.218
	CHO + MEN	33.89 (36.82, 37.17)	35.54 (35.27, 35.81)	35.44 (34.96, 35.91)	35.56 (35.15, 35.98)	*F*_(3,27)_ = 0.072, *p* = 0.974, $\eta_{p}^{2}$ = 0.008
Body temperature (°C)	CHO	35.80 (35.33, 36.27)	36.66 (36.44, 36.88)	36.92 (36.64, 37.20)	37.08 (36.66, 37.49)	*F*_(1,9)_ = 1.314, *p* = 0.281, $\eta_{p}^{2}$ = 0.127
	CHO + MEN	35.88 (35.68, 36.08)	36.76 (36.56, 36.95)	36.97 (36.67, 37.28)	37.18 (36.82, 37.55)	*F*_(3,27)_ = 0.026, *p* = 0.994, $\eta_{p}^{2}$ = 0.003
Heart rate (b min^−1^)	CHO	96 (85, 106)	137 (128, 146)	145 (135, 154)	177 (170, 183)	*F*_(1,9)_ = 3.557, *p* = 0.092, $\eta_{p}^{2}$ = 0.283
	CHO + MEN	91 (81, 102)	132 (123, 142)	139 (128, 151)	174 (166, 182)	*F*_(3,27)_ = 0.035, *p* = 0.991, $\eta_{p}^{2}$ = 0.004
PSI	CHO	‐	4.0 (3.7, 4.4)	5.0 (4.5, 5.5)	7.1 (6.7, 7.4)	*F*_(1,9)_ = 3.010, *p* = 0.117, $\eta_{p}^{2}$ = 0.251
	CHO + MEN	‐	3.8 (3.3, 4.3)	4.7 (4.0, 5.4)	6.9 (6.3, 7.4)	*F*_(2,18)_ = 0.040, *p* = 0.960, $\eta_{p}^{2}$ = 0.004
Percentage V̇O_2max_ (%)	CHO	‐	48 (45, 50)	50 (48, 53)	‐	*F*_(1,9)_ = 0.025, *p* = 0.878, $\eta_{p}^{2}$ = 0.002
	CHO + MEN	‐	47 (44, 50)	50 (47, 54)	‐	*F*_(1,9)_ = 0.560, *p* = 0.473, $\eta_{p}^{2}$ = 0.059
CHO utilisation (g min^−1^)	CHO	‐	1.91 (1.47, 2.34)	1.97 (1.60, 2.35)	‐	*F*_(1,9)_ = 0.966, *p* = 0.351, $\eta_{p}^{2}$ = 0.097
	CHO + MEN	‐	1.90 (1.49, 2.31)	1.95 (1.56, 2.34)	‐	*F*_(1,9)_ = 0.064, *p* = 0.806, $\eta_{p}^{2}$ = 0.007
FAT utilisation (g min^−1^)	CHO	‐	0.20 (0.13, 0.27)	0.22 (0.16, 0.29)	‐	*F*_(1,9)_ = 0.359, *p* = 0.564, $\eta_{p}^{2}$ = 0.038
	CHO + MEN	‐	0.22 (0.13, 0.33)	0.24 (0.15, 0.33)	‐	*F*_(1,9)_ = 0.012, *p* = 0.914, $\eta_{p}^{2}$ = 0.001
RPE^a^	CHO	10 (8–12)	12 (10–13)	12 (11–15)	19 (16–19)	*χ* ^2^ = 0.012, *p* = 0.914
	CHO + MEN	10 (8–12)	11 (10–14)	13 (11–16)	19 (17–19)	
Thermal sensation^a^	CHO	1‐ *Slightly warm* (1–2)	2‐ *Warm* (1–3)	2‐ *Warm* (1–4)	4‐ *Very hot* (3–4)	*χ* ^2^ = 0.108, *p* = 0.743
	CHO + MEN	1‐ *Slightly warm* (1–2)	2‐ *Warm* (1–3)	2‐ *Warm* (2–4)	4‐ *Very hot* (3–4)	
Thermal comfort^a^	CHO	1‐ *Comfortably warm* (0–1)	1‐ *Comfortably warm* (0–2)	1‐ *Comfortably warm* (1–3)	3‐ *Much too warm* (2–3)	*χ* ^2^ = 0.323, *p* = 0.570
	CHO + MEN	0‐ *Comfortable* (0–1)	1‐ *Comfortably warm* (1–2)	2‐ *Too warm* (1–3)	3‐ *Much too warm* (2–3)	

*Note*: Data are presented as mean (95% CI) with RM ANOVA outputs.

Abbreviations: CHO, carbohydrate; CHO + MEN, carbohydrate and menthol; PSI, physiological strain index; TT, time trial.

^a^ Analysed via Friedman nonparametric test and chi‐squared (*χ* ^2^) and *p* are presented.

There were no differences in the thermoregulatory data for rectal (CHO trial average: 37.63°C [37.59–37.68°C], CHO + MEN trial average: 37.60°C [37.55–37.66°C]), skin (CHO trial average: 35.04°C [34.96–35.12°C], CHO + MEN trial average: 35.25°C [35.16–35.33°C]) or body temperature (CHO trial average: 36.69°C [36.62–36.76°C], CHO + MEN trial average: 36.73°C [36.66–36.80°C]). In addition, there were no differences in the HR (CHO trial average: 139 b min^−1^ [138–140 b min^−1^], CHO + MEN trial average: 135 b min^−1^ [134–137 b min^−1^]) or end PSI (CHO: 7.1 [6.7–7.4], CHO + MEN: 6.9 [6.3–7.4]). Individuals worked at a similar percentage of V̇O_2max_ throughout the sub‐maximal exercise (CHO: 48% [45%–51%], CHO + MEN: 48% [44%–51%]).

### 3.3 Fifteen‐minute TT

**FIGURE 2:**
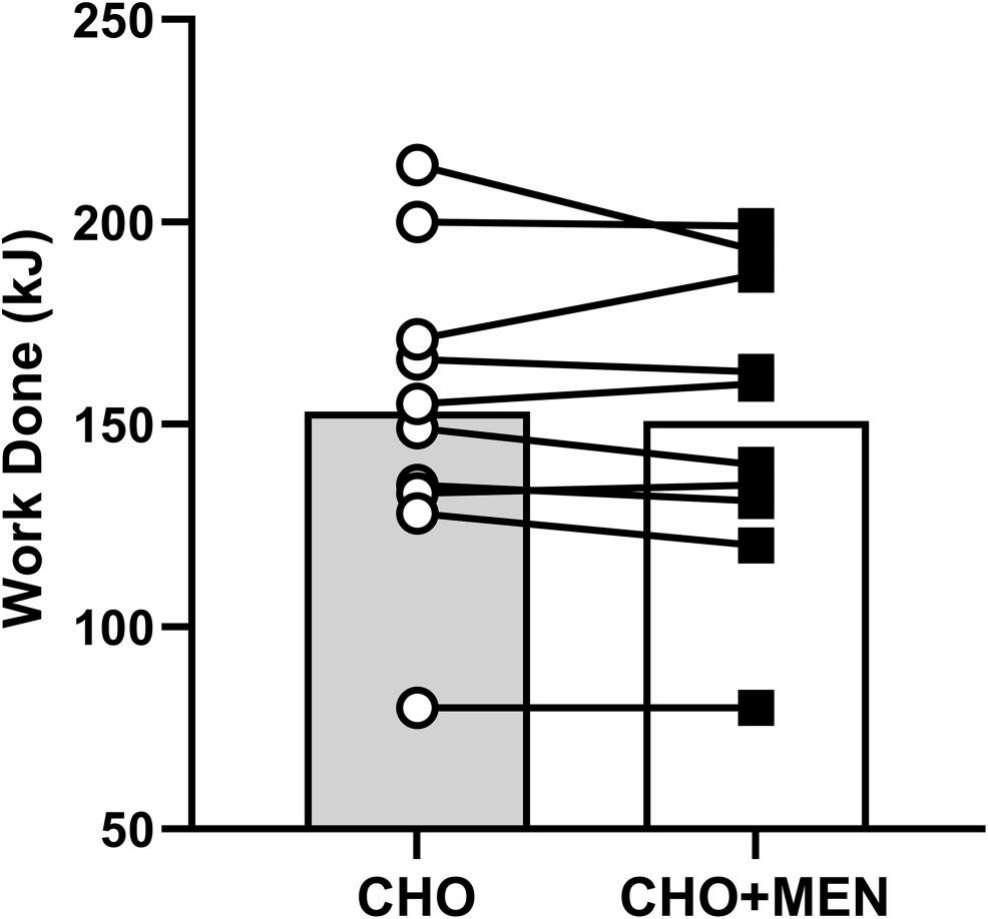
Work completed in the 15‐min TT for CHO and CHO + MEN condition. The bars are the group average, and lines are individual data. CHO + MEN, carbohydrate and menthol; TT, time trial.

No difference was seen between the work completed for CHO (153 kJ [129–177 kJ]) or CHO + MEN (151 kJ [128–174 kJ]), *p* = 0.472, *d =* 0.237, Figure 2.

### 3.4 Perceptions and follow‐up questionnaire

No difference was seen in TS (CHO: 4 [3, 4], CHO + MEN: 4 [3, 4]); TC (CHO: 3 [1, 3], CHO + MEN: 3 [2, 3]) or RPE (CHO: 19 [16, 19], CHO + MEN: 19 [17, 19]) (see Table 1).

**FIGURE 3:**
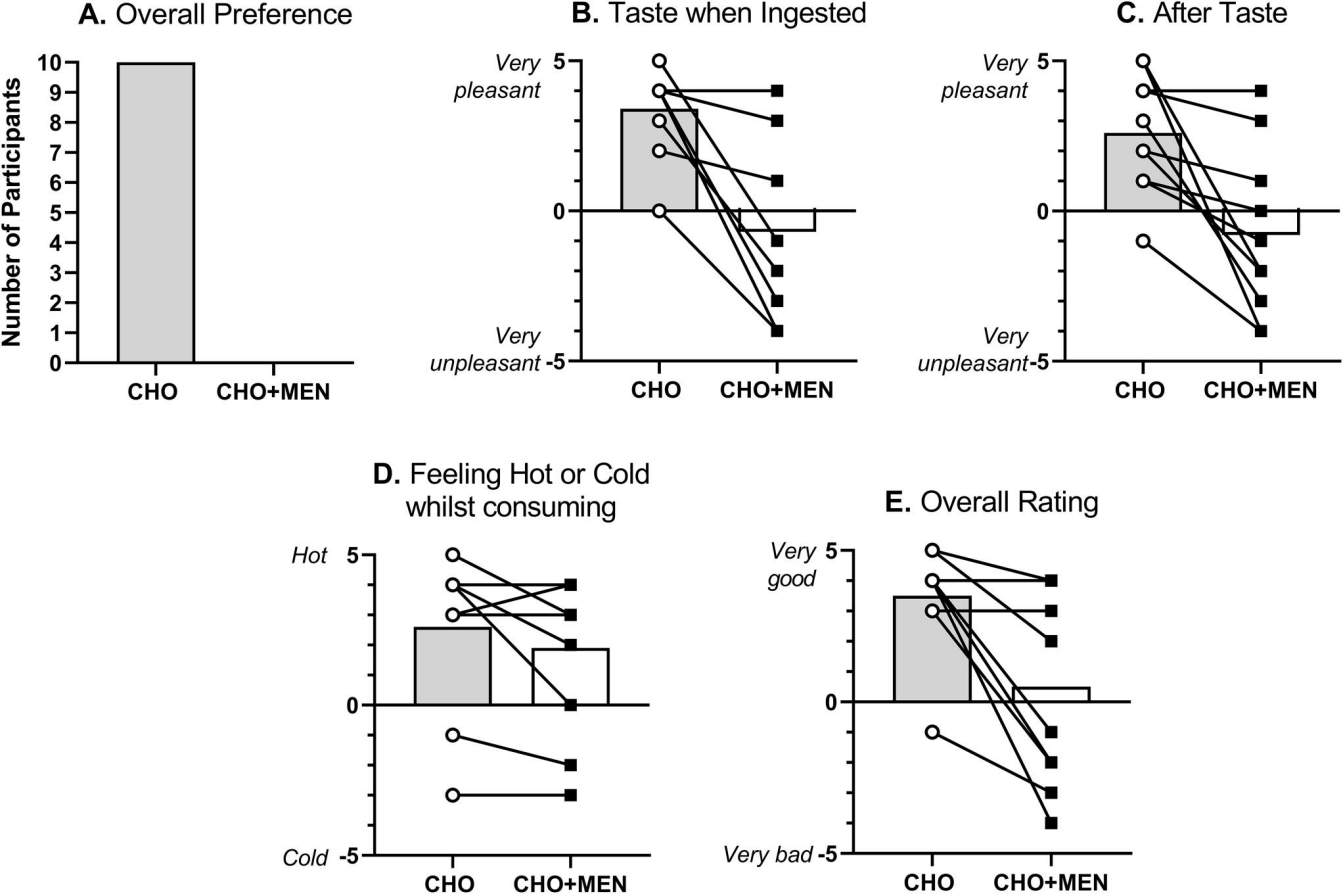
Follow‐up questionnaire responses for the CHO and CHO + MEN conditions. (A) Overall preference, (B) taste when ingested, (C) aftertaste, (D) feeling hot/cold when consumed and (E) overall rating. This questionnaire was completed whilst participants were blinded to the condition. CHO + MEN, carbohydrate and menthol.

However, all participants preferred the CHO drink compared with CHO + MEN (Figure 3A). When rating ‘taste ingested’, participants favoured the CHO drink (4 [0, 5]) versus CHO + MEN (−2 [−4, 4]) (*W* = 45.000, *p* = 0.009, Figure 3B). When rating ‘aftertaste’, participants favoured the CHO drink (3 [−1, 5]) versus CHO + MEN (−2 [−4, 4]) (*W* = 45.000, *p* = 0.009, Figure 3C). No difference was seen between ‘feeling of hot or cold sensation’ CHO (4 [−3, 5]) versus CHO + MEN (3 [−3, 4], *W* = 17.000, *p* = 0.202, Figure 3D). When rating overall experience, participants favoured the CHO drink (4 [−1, 5]) versus CHO + MEN (0 [−4, 4], *W* = 28.000, *p* = 0.022, Figure 3E).

## 4 DISCUSSION

The study showed that swilling and subsequent consumption of a commercial carbohydrate menthol drink had no effect on 15 min TT performance, perceptual or physiological variables compared with a commercial carbohydrate drink when exercising in the heat. The taste and aftertaste perceptions were favourable towards the carbohydrate only drink compared to the carbohydrate menthol drink.

No improvement was found in thermal perceptions or performance between the CHO + MEN and CHO drink, which contrasts with previous findings with isolated menthol in endurance exercise (Flood et al., 2017; Gavel et al., 2021). Oral menthol works as a perceptual cooling method by targeting the TRPM8 receptors within the oral cavity eliciting a cooling sensation, which in turn alters behaviour without altering physiological responses. In the current study, participant perceptions of TC and sensation were increased during exercise (i.e. feeling hot and uncomfortable); however, no differences were seen between conditions. Thermal perception has been shown to independently influence power output when conducting self‐paced exercise (Flood et al., 2017; Schlader et al., 2011). Menthol has the ability to reduce thermal perception whilst exercising in the heat, thus speculatively could have facilitated increased endurance performance (Flood et al., 2017; Gavel et al., 2021; Stevens et al., 2017, 2018). In addition of menthol to a carbohydrate drink had no effect on rectal temperature, skin temperature, HR or PSI during exercise. This is in keeping with the expected outcome that menthol would not alter rectal temperature (Mündel & Jones, 2010) or other physiological variables.

In contrast to previous studies, utilising menthol containing drinks, the commercial carbohydrate menthol drink in the present study had no additional effect compared to the carbohydrate drink on TC, sensation or RPE, neither did participants retrospectively rate any sensation of ‘coolness’. It is possible that the composition of the drinks used in the present study, which includes sweeteners, varying forms of carbohydrate (i.e. maltodextrin and dextrose), flavourings and electrolytes may have inhibited the distinct flavour and cooling sensation isolated menthol drinks have shown. This premise is supported by the participants rating both the taste and aftertaste of the carbohydrate menthol drink to be ‘unpleasant’, contrasting the ratings for the carbohydrate drink. This may have inhibited the positive cooling effect of menthol. Podlogar et al. (2021) used the same commercially available carbohydrate menthol drink as used in the present study and found similar findings with no alteration in TC or TS, and suggested it may be attributed to the composition of the drink. Interestingly, the aforementioned study found the combination of carbohydrate and menthol in a drink prompted a slight ‘cool’ feeling for participants, something not reported in the present study. However, the difference in ambient conditions (thermoneutral vs. hot humid) used by Podlogar et al. (2021) may have caused this difference. No performance improvement was found in either study, meaning that potentially the concentration of menthol within the CHO + MEN drink is insufficient to induce a cooling sensation and ultimately alter thermal perceptual measures. This is despite the carbohydrate menthol drink containing 0.01% menthol concentration in line with recommended concentrations that have shown perceptual and performance benefit in isolated drinks/swills (Jeffries & Waldron, 2019).

It is common for studies to investigate supplements in isolation in order to enhance possible effects (Podlogar et al., 2021). But in applied practice, supplements are rarely used individually, commonly consumed in combination with other supplements and often supplements effects become negligible (Burke, 2017). The use of isolated carbohydrate mouth rinsing as an ergogenic aid is well founded in improving performance without providing added energy to the individual (Carter et al., 2004). The exact mechanisms involved in carbohydrate rinsing are yet to be fully explained (Pomportes & Brisswalter, 2020); however, carbohydrate mouth rinsing stimulates areas of the brain that were key to reward and regulation of motor activity (Chambers et al., 2009). Although it is speculative, it may be that carbohydrate and menthol stimulation of oral receptors affect similar signalling route ways, inferring there is no additive effect of consuming both together (Podlogar et al., 2021).

Drink or rinse taste is known to convey useful information relating to a variety of factors, such as energy density, energy availability (such as CHO rinsing and consumption) and even food toxicity (Best et al., 2021; Breslin, 2013); specifically, taste reception relays signals to regions of the brain such as the primary taste cortex and putative secondary taste cortex in the orbitofrontal cortex (De Araujo et al., 2003; O’Doherty et al., 2001). From which, both the primary taste cortex and orbitofrontal cortex were believed to project to the dorsolateral prefrontal cortex, ventral striatum and anterior cingulate cortex (brain areas associated with autonomic and behavioural responses to stimuli of reward) (Rolls, 2007). Taste receptors were not limited to the oral cavity with the upper digestive tract also stimulated following ingestion (Best et al., 2021). Gam et al. (2014) showed rinsing and ingesting a bitter solution (quinine) improved cycling performance, whereas no improvement was found when just rinsing. Although not utilising menthol (which elicits a bitter taste), it raises an important distinction between mouth rinse and ingestion protocols. When considering carbohydrate and menthol in isolation, previous investigations highlight how performance improvements following carbohydrate rinsing are likely not related to taste (Best et al., 2021), shown by tasteless carbohydrate (e.g. maltodextrin) improved performance alongside activating similar brain regions associated with reward and motor control as sweet carbohydrates (Brietzke et al., 2019; Chambers et al., 2009). In addition, it has been shown that artificial sweeteners provide little brain activation despite the sweet taste (Frank et al., 2008). Therefore, the presence of carbohydrate rather than taste is likely sensed by oral receptors yet to be uncovered, inferring that in a carbohydrate menthol drink or rinse, potentially the taste of menthol may play a key role in activating brain activity (Best et al., 2021). Participants in the present study reported the ‘unpleasant’ and slightly bitter taste evoked in the carbohydrate menthol drink, however retrospectively rated no difference in ‘coolness’.

A recent study using menthol solution concentrations between 0.05% and 0.105% found that a stronger menthol concentration induced greater perceptual effects (feeling of ‘coolness’) (Best et al., 2018). In that study, concentrations of 0.95%–0.105% induced the greatest effect; however, some participants did not tolerate this concentration well. These data were supported by Stevens et al. (2021), where perceptual measures were assessed when consuming a combined carbohydrate menthol gel containing concentrations of 0.1% and 0.5% menthol. The study showed that strong concentrations were not well tolerated by all participants (despite evoking a greater ‘cool’ sensation). A flavoured menthol gel of varying concentrations (0.1%–0.7%) also evoked a cooling sensation; however, again it was not well tolerated by all participants, with higher concentrations causing greater irritability (Vogel et al., 2022). Potentially, the co‐ingestion of carbohydrate and other components within the drink may dull the effect of menthol suggesting that when consumed at the very low 0.01% concentration, there was no impact.

Drink or rinse taste is known to convey useful information to the brain relating to a variety of information such as energy density, energy availability (such as CHO rinsing and consumption) and even food toxicity (Best et al., 2021; Breslin, 2013). Taste receptors are not limited to the oral cavity, with the upper digestive tract also being stimulated following ingestion (Best et al., 2021). Gam et al. (2014) showed rinsing and ingesting a bitter solution (quinine) improved cycling performance, whereas no improvement was found when just rinsing. Although not utilising menthol (which elicits a bitter taste), it raises an important distinction between mouth rinse and ingestion protocols. When considering carbohydrate and menthol in isolation, previous investigations highlight how performance improvements following carbohydrate rinsing are likely not related to taste (Best et al., 2021), showing that tasteless carbohydrates (e.g. maltodextrin) improved performance alongside activating similar brain regions associated with reward and motor control as sweet carbohydrates (Brietzke et al., 2019; Chambers et al., 2009). In addition, it has been shown that artificial sweeteners provide little brain activation despite the sweet taste (Frank et al., 2008). Therefore, the presence of carbohydrate rather than taste is likely sensed by oral receptors yet to be uncovered, inferring that in a carbohydrate menthol drink or rinse, potentially the taste of menthol may play a key role in activating brain activity (Best et al., 2021). Participants in the present study reported the ‘unpleasant’ and slightly bitter taste evoked in the carbohydrate menthol drink, however, retrospectively rated no difference in ‘coolness’ and no change in TS/TC were seen.

Although blinding participants to the nature of the study was necessary to maintain a strong study design, it may have impacted upon participant performance. Without prior knowledge of the carbohydrate menthol drink, participants were not anticipating to feel ‘cooler’ as they might do in an applied setting, which may have altered individual performance outcomes (Podlogar et al., 2021). A notion accepted in sports ergogenics whereby psychological implications may occur if a participant believes they are receiving a ‘promising’ new product (Heneghan et al., 2012; Schulz et al., 2002). This is coupled with the research design masking the colour of drinks in opaque bottles. Best et al. (2018) found that solution colour influenced perceptual responses with light blue or green solution evoking the strongest responses to feeling ‘cool’.

Whilst the current study provides a valuable contribution on the understanding of co‐ingestion of carbohydrate and menthol, there are limitations. The project aimed to assess commercially available products; so, it assessed a traditional carbohydrate compared to a carbohydrate with menthol drink. By using these commercial products, there was no way to isolate menthol, that is, have a carbohydrate free approach, which could have provided additional insight. Instead, we were confident from recent meta‐analysis that menthol has been shown to improve perceptions during exercise in hot, humid conditions. In addition, no air flow was provided to participants in the current study, while it was thought that it would not change the outcome of the study (i.e. participants favouring the carbohydrate only drink), air flow may have contributed to the perception of cooling and could reduce the practical applications of the study.

In the current study, drink temperature was not measured throughout each trial. This is worth noting since drink temperature is known to affect the stimulation of oral TRPM8 receptors with cooler fluid eliciting a differing stimulation from TRPM8 receptors in comparison with tepid/warm fluid (Gavel et al., 2021). For all trials, drinks were made using tepid water, which was then placed into the environmental chamber 20 min before the trial started to minimise the impact of fluid temperature on stimulation. In addition, it is noted that habitual menthol use can impact TRPM8 stimulation; therefore, in future this should be assessed in each participant (Best et al., 2018). Future work should further investigate the co‐supplementation of carbohydrate and menthol as sports supplements are rarely used independently. However, we should consider having carbohydrate and menthol drinks separately and not be co‐ingested.

## 5 CONCLUSION

In conclusion, the ingestion of a commercial carbohydrate menthol drink did not influence physiological, perceptual variables or 15‐min TT performance in hot, humid conditions when compared with a commercial carbohydrate drink. The failure to alter perceptual responses or performance may be due to the co‐ingestion of menthol and carbohydrate inhibiting signalling pathways of menthol. In addition, all participants preferred the carbohydrate drink, and drink taste and after taste perceptions were favourable towards the carbohydrate drink compared to the carbohydrate menthol drink. The study suggests the mechanisms leading to the effects of menthol on exercise performance were potentially inhibited or that concentration is too low when co‐ingested with carbohydrate; and therefore, future research should look to separate rinses to identify future strategies for use in applied settings.

## CONFLICT OF INTEREST STATEMENT

There is no conflict of interest to declare.
